# Validation of an educational material to promote spiritual well-being in oncology patients

**DOI:** 10.15649/cuidarte.4726

**Published:** 2026-04-14

**Authors:** Barbara C. Salazar-Arce, Cielo Y. Pacho-Salinas, Daniel F. Condor-Camara

**Affiliations:** 1 Faculty of Nursing, Universidad Peruana Cayetano Heredia, Lima, Peru. E-mail: barbara.salazar.a@upch.pe Universidad Peruana Cayetano Heredia Lima Peru barbara.salazar.a@upch.pe; 2 Faculty of Nursing, Universidad Peruana Cayetano Heredia, Lima, Peru. E-mail: cielo.pacho.s@upch.pe Universidad Peruana Cayetano Heredia Lima Peru cielo.pacho.s@upch.pe; 3 Faculty of Nursing, Universidad Peruana Cayetano Heredia, Lima, Peru. E-mail: daniel.condor.c@upch.pe Universidad Peruana Cayetano Heredia Lima Peru daniel.condor.c@upch.pe

**Keywords:** Patient Education Handout, Teaching Materials, Health Education, Spirituality, Neoplasms, Folleto Informativo para Pacientes, Materiales de Enseñanza, Educación en Salud, Espiritualidad, Neoplasias, Prospecto para Educação de Pacientes, Materiais de Ensino, Educação em Saúde, Espiritualidade, Neoplasias

## Abstract

**Introduction:**

Cancer is a disease that impacts not only physical and emotional levels, but also the spiritual level. Therefore, spiritual well-being is essential for quality of life. In this context, educational materials designed to foster spiritual well-being play a crucial role.

**Objective:**

To validate educational material aimed at promoting spiritual well-being in oncology patients.

**Materials and Methods:**

A quantitative, descriptive, and cross-sectional study was conducted with exploration and validation phases of an educational material. The study population consisted of oncology patients and experts.

**Results:**

The target population showed a high level of spiritual well-being (26/30). The material was developed through an iterative review process. The validation and evaluation revealed that 7 out of 10 experts rated the educational material as superior, while 9 out of 10 members of the target population rated it as superior, particularly in the areas of “content,”“illustrations,” and “cultural appropriateness,” with a significant difference in “cultural appropriateness” (τ=0.73; p=0.03), where the target population perceived it more positively.

**Discussion:**

The material obtained high scores, and understanding spiritual well-being allowed the identification of improvements and adaptation of the material to the target audience. There is a lack of studies regarding the validation of educational materials on spiritual well-being in oncology. This study contributes to and encourages future culturally relevant research.

**Conclusion:**

The material was well accepted and developed according to the needs and literature, and it represents progress in promoting oncological care by providing an accessible and culturally appropriate tool.

## Resumen

**Introducción:**

El cáncer es una enfermedad que no solo tiene un impacto a nivel físico y emocional, sino también a nivel espiritual. Por ello, el bienestar espiritual es fundamental para la calidad de vida. En este contexto, los materiales educativos dirigidos a fomentar el bienestar espiritual adquieren un rol crucial.

**Objetivo:**

Validar un material educativo para promover el bienestar espiritual en pacientes oncológicos.

**Materiales y métodos:**

Estudio de enfoque cuantitativo, de diseño descriptivo y transversal con fases exploratorias y de validación de un material educativo. La población estuvo conformada por pacientes oncológicos y expertos.

**Resultados:**

La población objetivo presentó un alto nivel de bienestar espiritual (26/30). La elaboración del material se trabajó a través de una revisión iterativa. La validación y evaluación del material revelaron que 7/10 expertos calificaron el material educativo como superior, mientras que 9/10 miembros de la población objetivo lo evaluaron como superior especialmente en las áreas “Contenido”, “Ilustraciones” y “Adecuación cultural”, y significativo en la “Adecuación cultural” (τ=0,73; p=0,03), donde la población objetivo la percibe mejor.

**Discusión:**

El material obtuvo altas puntuaciones. Conocer el bienestar espiritual permitió identificar mejoras y adaptar el material al público objetivo. Faltan estudios sobre validación de materiales educativos en bienestar espiritual oncológico. Este trabajo aporta y motiva futuras investigaciones culturalmente pertinentes.

**Conclusiones:**

El material fue bien aceptado, desarrollado según necesidades y literatura, representa un avance en la promoción del cuidado oncológico al ofrecer una herramienta accesible y culturalmente pertinente.

## Resumo

**Introdução:**

O câncer é uma doença que impacta não apenas o bem-estar físico e emocional dos pacientes, mas também o bem-estar espiritual. Portanto, o bem-estar espiritual é fundamental para a qualidade de vida. Nesse contexto, materiais educativos que visam promover o bem-estar espiritual desempenham um papel crucial.

**Objetivo:**

Validar materiais educativos para promover o bem-estar espiritual em pacientes com câncer.

**Materiais e métodos:**

Este estudo utilizou uma abordagem quantitativa, delineamento descritivo e transversal, com fases exploratória e de validação para materiais educativos. A população foi composta por pacientes com câncer e especialistas.

**Resultados:**

A população-alvo apresentou alto nível de bem-estar espiritual (26/30). O material foi desenvolvido por meio de uma revisão iterativa. A validação e avaliação do material revelaram que 7/10 especialistas classificaram o material educativo como superior, enquanto 9/10 membros da população-alvo o classificaram como superior, especialmente nas áreas de "Conteúdo", "Ilustrações" e "Adequação Cultural", e significativamente em "Adequação Cultural" (τ = 0,73; p = 0,03), onde a população-alvo o percebeu melhor.

**Discussão:**

O material obteve pontuações altas. A compreensão do bem-estar espiritual permitiu identificar melhorias e adaptar o material ao público-alvo. Estudos sobre a validação de materiais educativos para o bem-estar espiritual oncológico são escassos. Este trabalho contribui e motiva futuras pesquisas culturalmente relevantes.

**Conclusões:**

O material foi bem aceito, desenvolvido de acordo com as necessidades e a literatura, e representa um avanço na promoção do cuidado oncológico por oferecer uma ferramenta acessível e culturalmente relevante.

## Introduction

Cancer is one of the most common causes of death worldwide. Among the most common types are breast, lung, colorectal, prostate, skin (non-melanoma), and gastric cancer^1^. For this reason, a holistic approach encompassing prevention through treatment is essential. In terms of global incidence, lung cancer ranks second, followed by breast cancer^2^.

The population between 50 and 75 years of age is more vulnerable to cancer, influenced by hereditary and external factors such as diet, sexual behaviors, alcohol consumption, exposure to ultraviolet radiation, chronic inflammation, and occupational exposure to chemical substances^3^.

After receiving a diagnosis, an individual may experience post-traumatic stress, which can affect them psychologically, physically, and spiritually^4^. During this process, oncology patients tend to focus on the disease and death, experiencing emotional disturbances influenced by factors such as personality, coping mechanisms, perceived social support, and economic and lifestyle constraints. These factors contribute to various reactive responses, manifested through significant behavioral changes^5^.

In addition to emotional well-being, oncologic diseases also affect the spiritual dimension, highlighting the importance of integrating spiritual care into treatment. This can be achieved through unbiased assessments and interventions designed to promote spiritual well-being^6^.

Spirituality is defined as a sense of inner peace connected to personal, social, and transcendent relationships with a higher entity or power^7^. It manifests through creative expression, rituals, meaningful relationships, purposeful activities, and religious practices, generating a perception of existential fulfillment^8^. This dimension directly influences cognitive development and both mental and physical health^6^,^9^, and it is recognized as a key factor in quality of life and well-being, encompassing beliefs about illness, the meaning attributed to it, and the inner peace it provides. Moreover, spiritual support contributes to managing stress, anxiety, and depression^10^,^11^, strengthens life purpose, and fosters a positive attitude toward treatment and recovery^4^. It plays a fundamental role in achieving an individual’s full health potential.

The assessment of spiritual well-being considers both beliefs and individual thoughts, addressing devotional and existential aspects to establish a sense of purpose and life satisfaction^12^. However, measuring spiritual well-being alone is not enough; it is essential to implement actions aimed at its improvement or maintenance. In this regard, various strategies such as counseling, consultations, and health education programs contribute to cognitive development.

These strategies generally fall under the responsibility of nursing professionals, who play a central role in patient education. Spiritual care is considered a fundamental dimension within the science of nursing care^9^. This reinforces the holistic approach that characterizes nurses as they take on multifaceted roles focused on the patient’s overall well-being.

Therefore, nursing professionals need to develop educational strategies supported by appropriate educational materials^6^. These resources are key to facilitating patient learning, promoting knowledge, and improving attitudes and skills^13^. The purpose of educational material lies in adapting to the needs of the target population^14^.

It is crucial to thoroughly monitor the target audience’s level of understanding, using questions to verify their comprehension. At the same time, it is essential to ensure that the language used is clear and concise, and to carefully assess the design and visual elements to optimize both understanding and the educational experience^15^. Educational materials are pedagogical tools that facilitate teaching and learning processes, and they include printed, technological, and digital resources^16^.

In this regard, the validation of educational material is fundamental as it positively contributes to the education of individuals and provides a valuable resource to address concerns when direct consultation with health personnel is not possible^15^.

The validation of educational material involves a process in which specific material is provided to a group of experts and members of the target population to evaluate and determine whether it fulfills the purpose for which it was designed. This process does not seek a “correct answer” but rather the creation of a resource tailored to the specific needs of patients^16^. Validated material provides coherent information and contributes to treatment adherence and informed decision-making by patients^17^.

The validation process defines what information should be included and evaluates whether the illustrations facilitate understanding of the content. Among the components to consider in this process are informational content, presentation, illustrations or graphics, language, stimulation or motivation elements, and cultural appropriateness^16^.

To ensure the suitability of educational materials, it is advisable to use instruments such as the Suitability Assessment of Materials (SAM)^18^ plus reliability and agreement assessment, which facilitate the assessment of the components mentioned. Furthermore, there are guidelines, such as those provided by the Pan American Health Organization (PAHO)^19^, which offer frameworks for the design, use, and evaluation of health education materials. These guidelines emphasize key aspects such as the use of clear and simple language, balanced image distribution, appropriate font and image sizes for better visualization, and strategic use of colors to ensure legibility and visual comfort.

In this context, this study aimed to validate an educational material designed to promote spiritual well-being in oncology patients.

## Materials and Methods

**Type of study:** A quantitative study with a descriptive and cross-sectional design was conducted, including exploration and validation phases of an educational material. The study was structured in three phases: [1] identification of the target population’s needs through an assessment of spiritual well-being; [2] development of educational material; and [3] validation and evaluation.

**Population: **The study enrolled oncology patients and experts in the field using a non-probabilistic convenience sampling technique. The literature indicates that between five and ten participants are sufficient to validate educational materials^15^,^20^.

In the first phase, all adult and older adult oncology patients from a private oncology clinic were invited during a scheduled visit. Inclusion criteria included the patient’s willingness to participate in the study, and exclusion criteria involved the lack of time or discomfort following treatment that prevented completion of the questionnaire. A total of 30 male and female patients participated, aged between 50 and 75.

The second phase did not include participants.

The third phase involved two groups: the first, consisting of experts—10 professionals, including physicians and nurses specialized in oncology—with more than five years of experience in the field as an inclusion criterion. The second group consisted of oncology patients—the target population—recruited after the development of the educational material. Once again, patients from the clinic were invited to take part, provided they had enough time to participate in the evaluation of the educational material. A total of 10 participants were recruited.

**Procedures:** In the first phase, each participant completed the* Meaning in Life Scale* (MiLS)^21^ questionnaire, which assessed their level of spiritual well-being. Some participants received assistance from a family member to complete the questionnaire.

In the second phase, the educational material was developed based on the results of the previous phase and a literature review that included books and scientific articles about inner peace, spirituality, and emotion management^8^,^22^-^25^. The design was produced by a graphic designer.

The third phase focused on the validation and evaluation of the educational material. For this purpose, the SAM instrument^18^ was used. Each participant received both the educational material and the instrument in printed format.

**Instruments:** Spiritual needs were evaluated using a modified version of the* Meaning in Life Scale* (MiLS)^21^,^26^ for Latin American populations, consisting of 21 questions distributed across four dimensions: [1] purpose and [2] level of meaning, each with seven questions rated on a Likert scale (1 = strongly disagree to 6 = strongly agree); [3] inner peace, with four questions; and [4] benefits of spirituality, with three questions, both dimensions rated on a Likert scale (0 = not at all to 4 = very much). For dimensions [3] and [4], the scale was redefined, increasing the maximum score from four to six points (0=1; 1=2.25; 2=3.5; 3=4.75; and 4=6). The total score was calculated by summing all item scores. A spiritual level of 70% or higher was considered high, while a level below that threshold was considered low or moderate.

The educational material was evaluated using the SAM instrument^18^, which provides a systematic method to determine the suitability of educational materials. This tool has been used in multiple studies^27^,^28^ and validated with a Content Validity Index (CVI) between 0.80^16^ and 0.99^28^, and internal consistency with Cronbach’s alpha of 0.91^28^. The instrument evaluates six areas: [1] content, [2]language, [3]illustrations, [4]presentation, [5]stimulation/motivation, and [6]cultural appropriateness. It consists of 22 questions rated on a Likert scale (0=not suitable, 1=adequate, 2=superior). Scores for subcategories in each area are summed and divided by the total possible score for that area, then multiplied by 10 for uniformity across factors. For the overall evaluation, all responses are summed. The scores obtained are converted into a percentage score, where “superior” = 70–100%, “adequate” = 40–69% and “not suitable” <39%^18^,^29^.

**Analysis Plan:** The analysis was performed in R v4.3.2. and RStudio v2023.12. Categorical data were presented as absolute frequencies, and numerical data as means, minimum, and maximum values. Kendall’s Rank Correlation Coefficient was used to measure concordance between expert and target population evaluations, with a significance level of p = 0.05. All collected data are available for open access and consultation on FigShare^30^.

**Ethical considerations:** The study was approved by the Institutional Ethics and Research Committee of the Universidad Peruana Cayetano Heredia, certificate No. 171-01–22, registration code: 206982. Data confidentiality was ensured, and all participants were informed in advance. All participants provided informed consent during the phases in which they participated. Authoriza-tion was also obtained from the* Unidad Oncológica Molecular Peruana *Health Center in Lima, Peru.

## Results

An educational material aimed at promoting spiritual well-being in oncology patients was validated through three phases, with the following results:

**Demographic characteristics of participants regarding spiritual well-being:** Twenty-two (22/30) participants were female, with a mean age of 62.50 years (ranging from 50 to 75 years). Nineteen (19/30) participants reported being Catholic. Regarding the time elapsed since disease diagnosis, sixteen (16/30) indicated a period between 1 and 11 months Table 1.

**Table 1:** Demographic characteristics of participants regarding spiritual well-being. n= 30

Characteristic	Frequency %(n)
Sex	
Male	26.67 (8)
Female	73.33 (22)
Religion	
Catholic	63.33 (19)
Christian	13.33 (4)
Letter - Day Saints	3.33 (1)
Worldwide Missionary Movement	3.33 (1)
Evangelical	3.33 (1)
None	13.33 (4)
Time since diagnosis	
1 month - 11 months	53.33 (16)
1 year - 5 years	33.33 (10)
5 years - 10 years	13.33 (4)
Age	
Mean (Standard Deviation)	62.50 ± 15.66
Minimum -Maximum	50-75

**Level of spiritual well-being:** Twenty-six (26/30) participants reported a high level of spiritual well-being. All assessed dimensions showed elevated levels of well-being, except for the inner peace dimension, which exhibited an even distribution among the evaluated levels Table 2.

**Development of educational material:** The content of the educational material was developed based on the results obtained in the previous phase and on a review of the literature^8^,^22^-^25^. In the “purpose” dimension, variables such as the level of personal fulfillment, perception of purpose in life, and satisfaction with daily activities were analyzed. The educational material included information on specific strategies designed to help patients identify and connect with their purposes, set meaningful goals, and maintain a positive attitude toward the future.

**Table 2:** Distribution of spiritual well-being levels. n= 30

Level	Frequency %(n)
Objective	
Low or moderate	13.33 (4)
High	86.67 (26)
Meaning	
Low or moderate	10.00 (3)
High	90.00 (27)
Inner peace	
Low or moderate	50.00 (15)
High	50.00 (15)
Benefits of spirituality	
Low or moderate	16.67 (5)
High	83.33 (25)
Total	
Low or moderate	13.33 (4)
High	86.67 (26)

In the “level of meaning” dimension, the focus was on the importance patients assigned to their lives and their motivation to achieve significant goals. The content for this dimension is centered on activities aimed at exploring and valuing sources of spiritual meaning, as well as promoting a rewarding and positive perspective.

In the “inner peace” dimension, the content focused on practical strategies to foster inner peace and emotional calmness, such as practicing compassion, active listening, and relaxation techniques.

Finally, in the “benefits of spirituality” dimension, the material included information on the role of spirituality in emotional and physical health, along with practical suggestions to cultivate confidence in healing and promote love for life.

**Figure 1:**
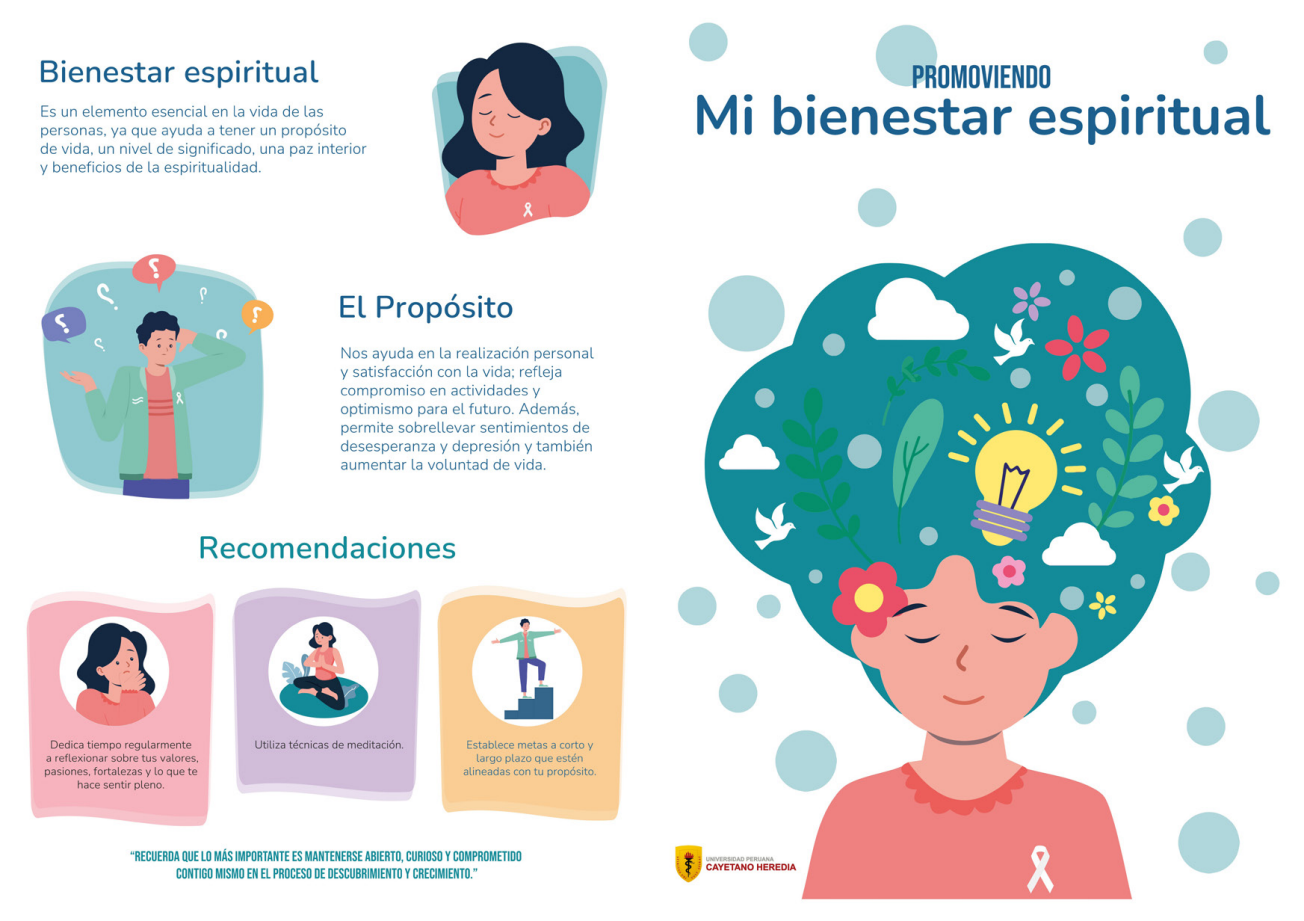
Educational material – front side: “Promoting my spiritual well-being”

**Figure 2:**
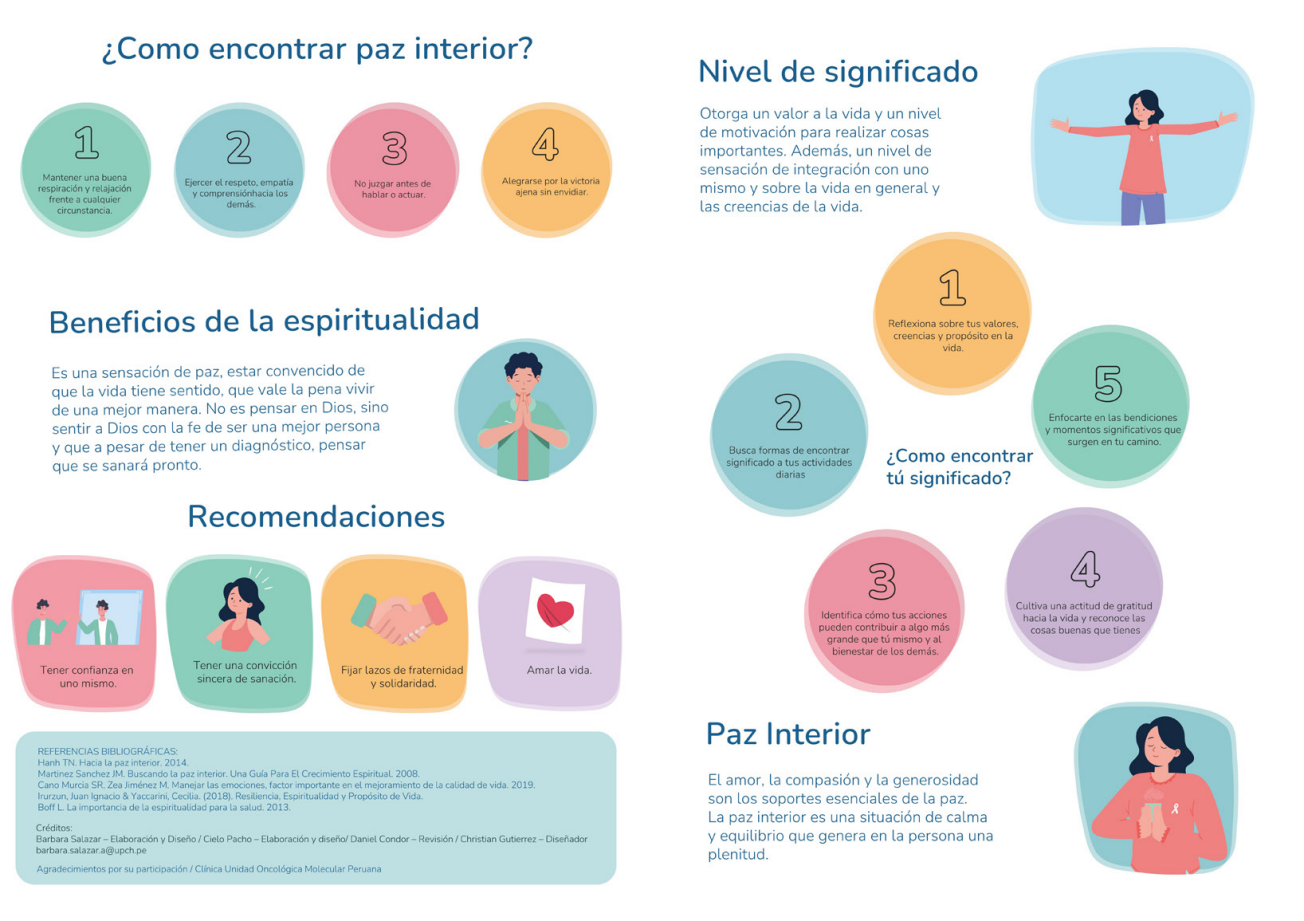
Educational material – back side: “Promoting my spiritual well-being”

The design of the educational material was produced by a graphic designer through an iterative review process in which feedback and recommendations were provided to improve the layout. Visual elements such as graphics and illustrations were incorporated to facilitate understanding of the content. The typefaces used were Bebas Neue Semi Rounded, Puck Medium, and Nud Motoya Maru Std-W5. Font sizes ranged from 14 to 32 points to adjust to the relevance of the text. The material was presented in an A4-sized leaflet format, divided into four sections to organize the information. For the purposes of the study, the material was utilized in the Spanish language Figures 1and 2.

**Validation and evaluation of educational material:** Both experts and the target population were predominantly female Table 3.

**Table 3:** Characteristics of participants in the validation and evaluation of the educational material

Characteristics	Experts n=10	Target population n=10
Sex		
Male	40.00 (4)	30.00 (3)
Female	60.00 (6)	70.00 (7)
Age		
Mean (SD)	35.50 (7.01)	61.90 (9.01)
Minimum Age-Maximum Age	25-45	52-75

SD: Standard Deviation

The evaluation of the educational material by the target population was more favorable compared to that of the experts, particularly in the areas of “content”, “illustrations”, and “cultural appropriateness”. The only statistically significant difference was found in “cultural appropriateness” (τ=0.73; p=0.03), where the target population rated it more positively. The overall evaluation suggested a moderate positive association, although not statistically significant (τ=0.51; p=0.13) Table 4. The comments and observations received were minimal; these were analyzed and promptly incorporated into the material, so a new validation round was not required.

**Table 4:** Evaluation of the educational material

Evaluation	Experts n=10	TP n=10	τ	p value*
Content				0.13
Adequate	50.00 (5)	20.00 (2)	0.50	
Superior	50.00 (5)	80.00 (8)		
Language				0.74
Adequate	10.00 (1)	10.00 (1)	-0.11	
Superior	90.00 (9)	90.00 (9)		
Illustrations				0.07
Adequate	20.00 (2)	10.00 (1)	0.67	
Superior	80.00 (8)	90.00 (9)		
Presentation				0.07
Adequate	40.00 (4)	20.00 (2)	0.61	
Superior	60.00 (6)	80.00 (8)		
Stimulation/motivation				0.07
Adequate	40.00 (4)	20.00 (2)	0.61	
Superior	60.00 (6)	80.00 (8)		
Cultural appropriateness				0.03
Adequate	10.00 (1)	- (0)	0.73	
Not suitable	10.00 (1)	10.00 (1)		
Superior	80.00 (8)	90.00 (9)		
Total				0.13
Superior	70.00 (7)	90.00 (9)	0.51	
Adequate	30.00 (3)	10.00 (1)		

TP: Target Population, τ = Kendall’s Tau (values closer to 1 indicate higher agreement), *Kendall’s Rank Correlation Coefficient

## Discussion

The study validated an educational material aimed at promoting spiritual well-being in oncology patients, where high scores were obtained. As a first step, the needs of the target population were identified. Participants demonstrated high levels of spiritual well-being, except in the “inner peace” dimension. This exception may relate to the emotional and spiritual burden associated with the illness, as well as the struggle with uncertainty, fear, and suffering. However, other studies indicate that individuals with advanced cancer often find positive meaning in stressful situations, which strengthens their spiritual perspective^31^-^33^.

The study also showed that most participants reported having a religious inclination. There is strong evidence of a relationship between religious spiritual well-being and resilience in oncology patients^8^,^34^. Additional studies have shown that allowing patients to express their religious beliefs during therapy reduces symptoms such as anxiety^35^ and positively influences coping with illness, including prostate cancer^36^. Nonetheless, this perspective can be complemented by broader approaches to spirituality that are not necessarily linked to religion^33^.

The development of the educational material was based on the identified needs of the target population and the review of the literature. The content was sequentially structured, clearly organized, and provided practical recommendations to achieve greater spiritual well-being.

The design contributed to organizing the information coherently^37^. The use of soft colors^38^, simple illustrations, and good readability facilitated visualization^19^, conveyed positive emotions^39^, and positively influenced content reception^40^.

The evaluation and validation process involved two stakeholder groups: subject-matter experts and the target population, ensuring objectivity in the evaluation. This process guaranteed accuracy, relevance, and cultural sensitivity in the development of material. Experts contributed credibility and helped identify areas for improvement to enhance the material’s impact^41^.

The perspective of the target population was essential, as it provided insights based on personal experience and helped determine whether the educational material addressed their needs, questions, and challenges. In addition, the evaluation included an assessment of the material’s religious sensitivity, clarity, and accessibility for older adults. Understanding the level of spiritual well-being was key to identifying areas for improvement and tailoring the material to the specific needs and concerns of the target audience^42^.

Although expert validation provides valuable academic and technical evaluation, it does not ensure accessibility, relevance, or usefulness for patients in real-world settings. Therefore, combining expert validation with feedback and direct experience from the target population is crucial to ensuring comprehensiveness and effectiveness of the educational material.

The study analyzed the agreement between experts’and participants’evaluations, revealing a tendency toward agreement, though not strong enough to assert a statistically significant correlation. This lack of correlation suggests the presence of divergent perceptions. While experts tend to evaluate from a technical and structural standpoint, patients prioritize emotional connection, clarity, and practical relevance. This difference highlights the need to balance technical criteria with real user experiences, so that the educational material is truly meaningful in clinical contexts^43^.

It is essential to highlight the lack of studies validating educational materials aimed at promoting spiritual well-being in oncology patients, which reveals a gap in the literature. This study contributes to partially closing that gap but also calls for future research studies to develop similar resources validated both technically and culturally.

Regarding limitations, validating educational material solely with experts may exclude the user’s perspective, introduce excessive abstraction due to academic specialization, result in insufficient practical validation, reduce sensitivity to cultural and religious aspects, and limit the evaluation of understandability and practical applicability^44^.

Furthermore, the study focused on a specific and small sample, limiting the generalization of the findings. However, there is no consensus on the ideal number to validate the content of an educational material^20^. It often depends on the desired level of experience and representation of the panel’s range of knowledge.

Another limitation was the absence of a formal adaptation of the SAM instrument into Spanish. Nevertheless, the instrument’s translation was done with the assistance of an English language professional, ensuring understanding among all participants.

## Conclusions

The study validated an educational material designed to promote spiritual well-being in oncology patients. The material received high acceptance from the target population. Its development was based on the identified needs and scientific literature, considering strategies to strengthen life purpose, meaning-seeking, inner peace, and the spiritual benefits of emotional and physical health.

Validation with both experts and the target population confirmed that content, illustrations, and cultural appropriateness were rated more positively by the target population compared to the experts. These findings highlight the importance of spiritual health as a key element in the comprehensive care of oncology patients.

The validated material represents a step forward in promoting cancer care by offering an accessible and culturally relevant tool. Its implementation in clinical practice would enable health professionals to effectively address the spiritual dimension of their patients.

Finally, although this study represents an initial contribution, future research should focus on validating instruments such as SAM in Spanish, expanding the sample to diverse clinical settings, and longitudinally assessing the material’s effectiveness in strengthening spiritual well-being. Finally, integrating the spiritual dimension into healthcare training would foster a more humane and person-centered approach.
